# The Severe Deficiency of the Somatotrope GH-Releasing Hormone/Growth Hormone/Insulin-Like Growth Factor 1 Axis of *Ghrh^−/−^* Mice Is Associated With an Important Splenic Atrophy and Relative B Lymphopenia

**DOI:** 10.3389/fendo.2018.00296

**Published:** 2018-06-06

**Authors:** Gwennaelle Bodart, Khalil Farhat, Chantal Renard-Charlet, Guillaume Becker, Alain Plenevaux, Roberto Salvatori, Vincent Geenen, Henri Martens

**Affiliations:** ^1^GIGA-I^3^ Center of Immunoendocrinology, GIGA Research Institute, University of Liege, Liège, Belgium; ^2^Cyclotron Research Center, University of Liege, Liège, Belgium; ^3^Division of Endocrinology, Diabetes, and Metabolism, Department of Medicine, The Johns Hopkins University, Baltimore, MD, United States

**Keywords:** somatotrope axis, GH-releasing hormone, growth hormone, insulin-like growth factor 1, thymus, developmental immunology

## Abstract

A debate is still open about the precise control exerted by the somatotrope GH-releasing hormone (GHRH)/growth hormone (GH)/insulin-like growth factor 1 axis on the immune system. The objective of this study was to directly address this question through the use of *Ghrh*^−/−^ mice that exhibit a severe deficiency of their somatotrope axis. After control backcross studies and normalization for the reduced global weight of transgenic mice, no difference in weight and cellularity of the thymus was observed in *Ghrh*^−/−^ mice when compared with C57BL/6 wild-type (WT) control mice. Similarly, no significant change was observed in frequency and number of thymic T cell subsets. In the periphery, *Ghrh*^−/−^ mice exhibited an increase in T cell proportion associated with a higher frequency of sjTREC and naïve T cells. However, all *Ghrh*^−/−^ mice displayed an absolute and relative splenic atrophy, in parallel with a decrease in B cell percentage. GH supplementation of transgenic mice for 6 weeks induced a significant increase in their global as well as absolute and relative splenic weight. Interestingly, the classical thymus involution following dexamethasone administration was shown to recover in WT mice more quickly than in mutant mice. Altogether, these data show that the severe somatotrope deficiency of *Ghrh*^−/−^ mice essentially impacts the spleen and B compartment of the adaptive immune system, while it only marginally affects thymic function and T cell development.

## Introduction

Integrated homeostasis of living organisms closely depends on the intimate crosstalk between the major systems of cell-to-cell signaling, the immune, nervous, and endocrine systems. Growth hormone (GH) secretion by somatotroph cells of the antehypophysis is stimulated by the hypothalamic GH-releasing hormone (GHRH) and its biological effects are mediated by GH receptor, a member of the class I cytokine receptor superfamily (1). Most of the metabolic effects of GH on peripheral tissues are direct, while the growth-promoting action is mainly indirect, through the endocrine and paracrine–autocrine insulin-like growth factor 1 (IGF1).

Already in 1930, Philip E. Smith observed that hypophysectomy in rats induced a severe thymic involution (1). Numerous studies in two mouse models of pituitary deficiency, the Snell–Bagg and Ames Dwarf mice—mutated in Pit1 and Prop1 transcription factors, respectively—confirmed the regulation of the immune system by pituitary hormones since those mice exhibited a thymo-dependent immune deficiency that could be prevented or reversed by administration of GH and thyroxine (2–4). This was reinforced by the observation of aged-atrophic thymus rejuvenation by GH-producing pituitary adenoma cells (5). The effects mediated by GH and IGF1 on immune cells have been extensively reviewed (6, 7).

Positive effects of the somatotrope axis on human thymopoiesis have also been reported. Treatment of HIV+ patients with GH in combination with highly active antiretroviral therapy increases thymic volume, CD4^+^ naïve T cell number, and thymopoiesis evaluated by the frequency of signal joint (sj) T-cell receptor excision circles (TRECs) (8). One-month withdrawal of GH in patients with adult GH deficiency diminished thymic output of new T cells and intrathymic T-cell proliferation, as evidenced by the decrease in sjTREC frequency and sj/DJβTREC ratio, respectively, and these parameters were restored 1 month after GH resumption (9). Given their important beneficial effects on thymopoiesis, GH and IGF1 are more and more considered for their use as immunomodulatory agents in acquired immune deficiencies such as in HIV infection and aging.

Despite this large experimental evidence, the role of GH in immunologic regulation remains controversial and is still discussed. In contradiction with previous studies, some authors observed a normal thymus weight and number of T-cell subsets in Snell–Bagg dwarf and in *lit/lit* (GHRH receptor-deficient mice) mice (10–12). Dorshkind and Horseman (13) concluded that the immunomodulatory properties of the pituitary hormones GH and prolactin essentially result from their ability of counteracting the negative effects mediated by stress-induced glucocorticoids (GCs) upon the immune system.

The objective of this study was to investigate the basal thymic and immunologic phenotype of a transgenic mouse model with a severe deficiency of the somatotrope axis resulting from a targeted disruption of the GHRH gene (14). *Ghrh*^−/−^ mice exhibit a dwarf phenotype due to a severe GH and IGF-1 deficiency that can be supplemented either at the hypothalamic, hypophysial, or peripheral levels of the somatotrope axis (15–17).

## Materials and Methods

### Mice

*Ghrh*KO mouse strain (C57BL/6 background) was previously developed by one of us as previously described (14). Wild-type (WT) C57BL/6 mice were obtained from Charles River Laboratories. Both strains were kept and bred at the animal facility of the University of Liège. All animals were bred in ventilated cages at the Central Animal Facility of Liege University (GLP certified, LA.2610359) of the University of Liège with a 12-h light/12-h dark cycle with food and water *ad libitum*. We performed a backcross between those two strains, to obtain animals with completely identical genetic background. Briefly, *Ghrh*KO and C57Bl/6 mice were bred together to obtain a F1 generation of heterozygous (HZ) animals. F1 animals were mated together and gave rise to F2 mice with *Ghrh^+/+^* (called WT in the text), *Ghrh^+/−^* (HZb), and *Ghrh*^−/−^ (called *Ghrh*KO in the text) animals (respectively, 25, 50, and 25% proportion expected). Mouse genotype was identified phenotypically: original *Ghrh*^−/−^ mice have agouti color, a dominant trait, where agouti gene is located near the *Ghrh* mutated gene, so they are transmitted together. Therefore, WT backcrossed F2 mice are black and normal-sized; HZ backcrossed animals are agouti and normal-sized and KO backcrossed mice are agouti and dwarf. Normal-sized and dwarf mice were separated at least 4 weeks before any experiment. Male and female mice of 3, 6, or 18 months were used for the characterization experiments, and 3 or 18 months for the GH supplementation experiments. All the experiments were conducted with approval of the Institutional Animal Care and Use Committee of the University of Liège (permit no. 1305) in strict accordance with the guidelines for the care and use animals set out by the European Union.

### Tissue and Cell Preparation

Mice were euthanized by i.p. injection of ketamine (100 mg/kg)–xylazine (10 mg/kg) followed by cardiac puncture. Thymus, spleen, and inguinal lymph nodes (LNs) were removed and weighted. A piece of liver was also removed when needed for IGF-1 quantification. PBMC were isolated from whole blood by centrifugation in Lympholyte^®^-Mammal density separation medium (Cedarlane), according to the manufacturer’s instructions. Single-cell suspensions were obtained from the thymus, spleen, and LN by mechanical disruption, followed by two washing steps at 500 *g* for 5 min in Dulbecco’s phosphate-buffered saline (DPBS, Lonza). An additional RBC lysing step was performed to eliminate RBC from splenic cell suspension by incubating 5 min in 1 ml of RBC Lysis Buffer Hybri-Max (Sigma-Aldrich) before a final washing step. Cell suspensions were then passed through 70-µm Nylon cell strainer (Falcon) and diluted to the appropriated concentration in DPBS.

### Flow Cytometry

For analysis of lymphocyte subpopulations in thymus, blood, spleen, and LN, cells were stained with the following mAbs: anti-mouse CD45.2 FITC (clone 104), CD19 Brilliant Violet 510 (1D3), CD44 APC (IM7), CD62L PE (MEL-14) were purchased from BD Biosciences. Anti-mouse CD4 eFluor^®^450 (RM4-5), CD8a Pe-Cyanine7 (53-6.7), CD90.2 (Thy-1.2) APC (53-2.1), and Foxp3 PE (FJK-16s) were purchased from eBioscience.

Cells were counted in Neubauer Chamber and approximately 500,000 cells were used for flow cytometry analysis. Briefly, cells were washed in DPBS and labeled with a cocktail of mAbs specific for cell surface Ag diluted in DPBS containing 2% FBS. After 20 min incubation at 4°C in the dark, labeled cells were washed in DPBS containing 2% FBS and resuspended in DPBS before analysis. For Foxp3 intracellular staining, cells were labeled for surface Ag, washed in DPBS, fixed, and permeabilized with fixation/permeabilization solution (Anti-Mouse/Rat Foxp3 Staining Set, eBioscience) according to the manufacturer’s instructions and stained for intracellular Foxp3. Labeled cells were analyzed on a BD FACS Verse (BD Biosciences) using BD FACS Suite Software (BD Biosciences). Number of cells was calculated in function of the volume of cell suspension analyzed by the FACS Verse and multiplied by the dilution factor and the factor of proportion of cell suspension used for flow cytometry compare to the total volume of suspension.

### TREC Quantification

PCR quantification (qPCR) of sjTREC and DJβTREC were performed according to a protocol adapted from Dulude et al. (18), using CD4 gene as a reference single-copy gene. Briefly, cells were lysed in lysing buffer containing Tris–HCl (10 mM; pH 8.3), Tween 20 (0.05%), Igepal (0.05%), and proteinase K (100 µg/ml) for 30 min at 56°C followed by proteinase K inactivation (10 min at 95°C). DNA from cell lysates was preamplified in an iCycler (Bio-Rad) using outer primers (Table 1) and GoTaq^®^ Flexi DNA Polymerase (Promega) with the following conditions: initial denaturation at 95°C for 10 min; 22 cycles of amplification at 95°C for 30 s; 60° for 30 s; 72°C for 2 min; final elongation 72°C for 10 min; and cooling at 15°C. In this first-step of amplification, CD4 gene was coamplified together with the sj- or DJβTREC. Plasmids containing CD4 and sj61 or DJβ4 sequences were preamplified in the same way and used to generate standard curves. PCR products were diluted and CD4 and TREC amplicons were quantified by qPCR in a LightCycler480 thermocycler (Roche Diagnostics) using Takyon™ No Rox SYBR MasterMix Blue dTTP (Eurogentec) and inner primers (Table 1) with the following conditions: 5 min of initial denaturation at 95°C; 40 cycles of amplification at 95°C for 10 s; 60°C for 15 s; 72°C for 10 s; and cooling at 40°C. Results were analyzed on the LightCycler480 Software and expressed in number of TREC per 10^6^ cells. All probes were purchased from Eurogentec. Total sjTREC content was estimated by multiplex quantification of δREC1/jα61 and δREC1/jα58 rearrangements, since they account for almost 100% of total sjTREC frequency (18). Similarly, dβTREC content was obtained by multiplex nested-PCR of dβ1 rearrangement with Jβ1.1-1.6. An internal control was added to each run of qPCR to evaluate the run-to-run variation. When the SD for the control sample was above 10%, a correction factor (TREC content of control sample in this run/mean TREC content of control sample for all run) was applied to each sample of the run.

**Table 1:** Outer and inner primers for T-cell receptor excision circle quantification.

Name	Sequences out	Sequences in
CD4 1	CCAACCAACAAGAGCTCAAGGA	AGCTCAAGGAGACCACCATGT
CD4 2	CCCAGAATCTTCCTCTGGT	TGGTCAGAGAACTTCCAGGT
Jα61	AACTGCCTGGTGTGATAAGAT	GGAGTATCTCTTTGGAGTGA
Jα58	CCCAGGACACCTAAAAGGAT	AACTCGCACAGTGGAGGAAA
REC1	AGTGTGTCCTCAGCCTTGAT	GAAAACCTCCCCTAGGAAGA
dβ1	TATCCACTGATGGTGGTCTGTT	GACGTTGGCAGAAGAGGATT
Jβ1.1	CATGTTTGACATTGCCACAAGT	AGCGATTACTCCTCCTATGGT
Jβ1.2	CTCTCTTCACCCCTTAAGATT	GTAAAGGAACCAGACTCACAGTT
Jβ1.3	TGAGGCTGGATCCACAAAGGT	TCAAGATGAACCTCGGGTGGA
Jβ1.4	GGGCCATTAGGAAACGTGAT	GCAGGAAGCATGAGGAAGTT
Jβ1.5	GGAGGAAGGAAGGATGGTGA	CAGAGTCCTGCCTCAAAGAA
Jβ1.6	CCTGTGACATGCCTCATGGTA	TCAGGTCTCAGGGATCTAAGA

### GH Supplementation

Mice were daily injected i.p. with either human recombinant GH (1 mg/kg in 100 µl DPBS, Genotonorm, Pfizer) or DPBS as control for 6 weeks. Two weeks before injection, a blood sample was taken from the tail for TREC quantification. At day 0 before injection and once per week after the beginning of the treatment, glycemia and weight were measured to follow the effect of GH treatment and a blood sample (130 µl in WT and 65 µl in KO mice) was taken from the tail weekly for flow cytometry analysis or TREC quantification (each analysis was alternatively performed every second week). After 6 weeks of treatment, mice were euthanized by i.p. injection of ketamine (100 mg/kg)–xylazine (10 mg/kg) followed by cardiac puncture and thymus, spleen, LN, and liver removal.

### *Igf1* Quantification by Real-Time Quantitative PCR

*Igf1* transcripts were analyzed as previously described (19). RNA extraction was performed using NucleoSpin^®^ RNA kit (Macherey-Nagel) according to the manufacturer’s instructions. Liver tissue extracts kept at 4°C in RNAlater (Qiagen) and thymic cell suspensions were disrupted in lysis buffer containing β-mercaptoethanol and stored at −80°C until RNA extraction. After extraction, RNA concentration was measured by NanoDrop ND-1000 (Thermo Scientific) and 500 ng were used for reverse-transcription with oligo-dT using Transcriptor first strand cDNA synthesis Kit (Roche). Quantitative PCR was performed in the iCycler (Bio-Rad) using Taqman probes technology and iQSupermix (Bio-Rad) with the following primers: *Igf1* forward CAGGCTATGGCTCCAGCATT; *Igf1* reverse ATAGAGCGGGCTGCTTTTG; probe 6-FAM-AGGGCACCTCAGACAGGCATTGTGG-BHQ-1. Mouse hypoxanthine–guanine phosphoribosyltransferase (HPRT, Mm01324427_m1 TaqMan Gene Expression Assays, Applied Biosystems) in the following conditions: polymerase activation at 50°C for 2 min; preliminary denaturation at 95°C for 10 min; 50 cycles of amplification 95°C for 15 s; 60°C for 1 min. Mouse *Hprt* was used as a housekeeping gene. Number of copies for *Hprt* and total *Igf1* were calculated from the linear regression of standard curve generated from serial dilution of plasmids specifics for each gene.

### Dexamethasone (DXM) Administration

Mice were injected i.p. with 20 mg/kg Dexamethasone dihydrogenophosphat-Dinatrium (Aacidexam 5 mg/ml, Aspen) or DPBS as control. The day of injection was referred to as d0. MRI sessions were performed at day 0, 2, 5, 10, and 14 to follow thymic involution and recovery. At d15, mice were euthanized and thymus, blood, spleen, and LN were removed for further analysis.

### MRI Data Acquisition and Processing

Anesthesia was induced with isoflurane 4% in air, and then maintained by reducing the ratio to 1.5% for the duration of the acquisition (flow rate: 0.8 l/min). The mice were placed prone in a stereotaxic holder (Minerve, France). The breathing rate was monitored during the entire scan and the body temperature maintained at 37 ± 0.5°C with an air warming system (Minerve, France). MRI anatomical images were acquired on a 9.4 T MRI DirectDrive VNMRS horizontal bore system with a shielded gradient system (Agilent Technologies, Palo Alto, CA, USA) and a 40-mm inner diameter volumetric coil (Agilent Technologies, Palo Alto, CA, USA). Fast spin echo multislices sequence were acquired using the following parameters adapted from Brooks et al. (20) and Beckmann et al. (21): TR/TE_eff_ = 2,000/40 ms, matrix = 192 × 192, FOV = 20 mm × 25 mm, 10 contiguous slices focused on the region of interest (thickness = 1.0 mm, in-plane voxel size: 0.104 mm × 0.130 mm). Anatomical images were analyzed using PMOD software version 3.6 (PMOD Technologies Ltd., Zurich, Switzerland). The thymus was manually segmented, thanks to its difference in signal intensity from the surrounding tissues, on each contiguous slice (thereafter refer as region-of-interest, ROI). The PMOD tools allow direct computing of the organ volume, by multiplying the effective slice thickness with the surface areas of each ROI.

### Statistical Analysis

Statistical analyses were performed on the Prism 4.0 software (GraphPad). Kolmogorov–Smirnov and Shapiro–Wilk normality tests were performed to evaluate the Gaussian distribution of results. Unpaired *t*-test was applied when Gaussian distribution was verified, and Mann–Whitney test for non-Gaussian distributions. For multi-parametric analysis of GH supplementation, two-way ANOVA with Bonferroni post-test was used.

## Results

### Somatotrope Deficiency in GhrhKO Mice Affects Lymphoid Organ Weight and Cellularity

As previously described (14), *Ghrh*KO mice have a dwarf phenotype, with an adult weight 50% smaller than control counterpart (Table 2 and Table S1). Female but not male mutant mice catch up weight of WT mice with age, but it is mainly due to fat accumulation (personal observation). Both spleen and thymus are smaller in *Ghrh*KO mice (Table 2 and Table S1). When corrected to total body weight, the spleen remains proportionally smaller in mutant mice (about 40% reduction). On the opposite, the relative thymus weight is similar between normal and mutant mice and decreases at 18 months of age in both strains (Table 2 and Table S1). Since the absolute weight in *Ghrh*KO thymus is stable with time, unlike WT mice, this decrease in relative weight could not be attributed to thymic atrophy, but rather to the significant increase in total body weight.

**Table 2:** Effects of somatotrope deficiency on weight and number of cells of lymphoid organs.

	3 Months		6 Months		18 Months	
			
	C57BL/6 wild-type (WT) (*n* = 3♂ 12♀)^c^	*Ghrh*KO (*n* = 10♂ 6♀)^c^	C57BL/6 WT (*n* = 2♂ 6♀)^b^	*Ghrh*KO (*n* = 4♂ 12♀)^c^	C57BL/6 WT (*n* = 3♂ 6♀)^c^	*Ghrh*KO (*n* = 1♂ 5♀)^a^
**Body weight (g)**						
Male ♂	24.5 ± 0.18	13.1 ± 0.18***	32.0 ± 1.15^(a)^	16.7 ± 0.41^***(a)^	33.6 ± 5.01	15.9
Female ♀	21.3 ± 0.36	11.8 ± 0.14***	24.4 ± 0.75^(a)^	15.9 ± 0.54^***(a)^	27.0 ± 0.23^(a,b)^	24.7 ± 2.26^(a,b)^
**Thymus**						
Absolute weight (mg)	53.2 ± 4.28	29.6 ± 2.56***	58.6 ± 5.61	29.62 ± 1.87***	23.4 ± 2.78^(a,b)^	29.9 ± 2.40
Relative weight (mg/g of body weight)	2.5 ± 0.24	2.2 ± 0.25	2.3 ± 0.26	1.7 ± 0.12	0.9 ± 0.12^(a,b)^	1.2 ± 0.12^(a,b)^
Absolute number of cells (×10^6^)	47.3 ± 6.11	32.4 ± 4.56*	35.5 ± 3.14	20.8 ± 1.82^*(a)^	16.3 ± 7.90^(a)^	ND
Relative number of cells (×10^4^/mg of thymus)	84.3 ± 15.76	107.9 ± 18.00	62.7 ± 5.58	76.3 ± 7.93	38.7 ± 12.27^(b)^	ND
**Spleen**						
Absolute weight (mg)	79.1 ± 3.19	31.0 ± 2.04***	77.7 ± 9.4	35.6 ± 1.86***	97.4 ± 4.51^(a)^	59.2 ± 8.19^***(a,b)^
Relative weight (mg/g of body weight)	3.6 ± 0.15	2.2 ± 0.06***	3.1 ± 0.43	2.2 ± 0.10**	3.4 ± 0.25	2.2 ± 0.19**
Absolute number of cells (×10^6^)	44.8 ± 4.61	20.3 ± 2.05***	51.3 ± 6.08^(a)^	18.1 ± 1.78***	21.5 ± 3.18^(a,b)^	18.5 ± 3.68
Relative number of cells (×10^4^/mg of spleen)	56.7 ± 5.11	63.6 ± 3.53	52.5 ± 3.79	50.2 ± 4.25^(a)^	21.5 ± 2.22^(a,b)^	34.8 ± 7.36^(a)^

*Data (mean ± SEM) are representative of one^a^, two^b^, or three^c^ independent experiments*.

*ND, not determined*.

Unpaired *t*-test or Mann–Whitney test were used according to the Gaussian distribution of each set of data. Significant difference from age-matched WT mice ***p < 0.001, **p < 0.01, *p < 0.05; significant difference compared to 3-^(a)^ or 6-^(b)^month-old mice from the same strain.

The absolute number of CD45^+^ leukocytes in spleen and LNs and CD90.2^+^ (Thy1.2) thymocytes in thymus are also reduced in *Ghrh*KO mice compared to WT mice (Table 2 and Table S1). When corrected to the weight of the corresponding organ, they are no differences in relative cellularity between both strains (Table 2 and Table S1). Both mutant and normal mice undergo a loss of cell number and density with age in the two organs. Moreover, thymic involution is clearly evidenced by the age-dependent loss of the absolute thymocyte number in both strains. Loss of cells is accompanied by reduction in tissue weight in 18-month-old C57BL/6 mice. On the contrary, thymic T-cell disappearance in *Ghrh*KO mice seems to be equally compensated by fat or other tissue as thymus absolute weight does not vary with time.

### Somatotrope Deficiency Is Associated With Minor Changes in Thymocytes Subsets Repartition

**Figure 1:**
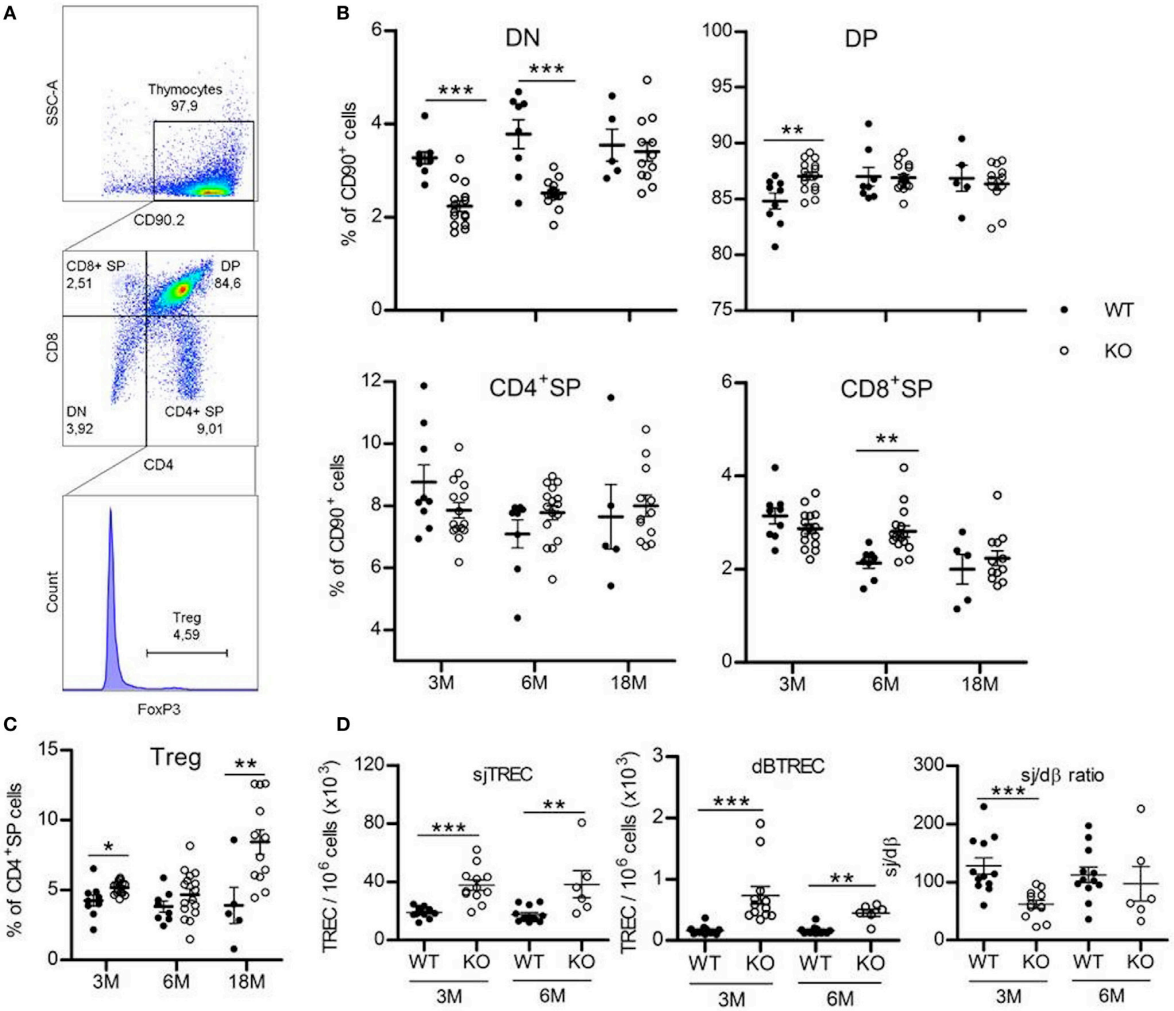
Thymus phenotype and function. **(A)** Representative flow cytometry plots showing the four thymic subpopulations double-negative (DN) (CD4^−^CD8^−^), double-positive (DP) (CD4^+^CD8^+^), CD4^+^ single-positive (SP) (CD4^+^CD8^−^), and CD8^+^ SP (CD4^−^CD8^+^) cells, identified within the CD90.2^+^ population. FoxP3^+^ Treg cells are studied inside the CD4^+^ SP population. 30,000 events are recorded. **(B,C)** Frequencies of the four thymic T-cell subpopulations **(B)** and FoxP3^+^ Treg cells **(C)** in thymus of KO (white circles, *n* = 12–16) and wild-type (WT) (black circles, *n* = 5–9) mice at 3, 6, or 18 months. Data (mean ± SEM) are representative of two to three independent experiments. Unpaired *t*-test was used for statistical analysis. **(D)** sjTREC, dβTREC, and sj/dβ ratio in splenocytes of KO (white circles, *n* = 12) and WT (black circles, *n* = 6–12) mice at 3 and 6 months are shown. Data (mean ± SEM) are representative of two independent experiments. Unpaired *t*-test was used for statistical analysis. ***p < 0.001, **p < 0.01, *p < 0.05.

Thymopoiesis is first assessed by analyzing thymocyte phenotype. Thymic T-cells are subdivided in four subpopulations based on their expression of CD4 and CD8 surface molecules. The most immature CD4^−^CD8^−^ subset is called double-negative (DN) cells. They evolve and acquire expression of both CD4 and CD8 to become double-positive (DP) cells, then mature to single-positive (SP) CD4^+^ or CD8^+^ cells by losing expression of either CD8 or CD4 molecules, respectively (Figures 1A,B; Table S2 in Supplementary Material). Flow cytometry analysis of thymic T-cell subpopulations shows a significant decrease (about one-third) in frequency of DN subset in *Ghrh*KO compared with C57BL/6 mice (WT: 3.3 ± 0.13% vs KO: 2.2 ± 0.12% at 3 months and WT: 3.8 ± 0.31% vs KO: 2.5 ± 0.07% at 6 months), compensated by an increase in DP cells at 3 months (84.8 ± 0.70% for WT vs 87.0 ± 0.36% for KO) and CD8^+^ SP cells at 6 months (2.1 ± 0.11% for WT vs 2.8 ± 0.12% for KO; Figure 1B; Table S2 in Supplementary Material). At 18-month-old, frequency of DN cells in mutant mice increases compare to younger animals, to be equal to values in normal mice (WT: 3.4 ± 0.20% and KO: 3.55 ± 0.34%). In addition, Treg cells in CD4^+^ SP subset (Figure 1C; Table S2 in Supplementary Material) increases in thymus of mutant mice (*p* = 0.011 for 3 months, *p* = 0.208 for 6 months, and *p* = 0.013 for 18 months).

Taken together, those results could indicate a faster commitment of *Ghrh*KO DN cells in the thymopoietic process, leading to lower frequency of the most immature thymic T-cells and increasing number of the subsequent stages of development. Finally, at advanced age (18 months), thymocyte frequency and number return to values equivalent to WT mice.

### TREC Numbers Are Increased in GhrhKO Mice

A widespread method to assess thymic function is the quantification of TREC, the small circles of DNA produced during TCR rearrangement of segment genes coding for α and β chains of the T-cell receptor for the antigen (TCR). They offer the advantage to be stable in cells and as duplicated during mitosis, slowly diluted by cell proliferation. dβTREC are created at an early stage of thymopoiesis during β chain rearrangement in DN cells and sjTREC are products of δ locus deletion in DP and SP cells. Therefore, sjTREC are present in almost all recent thymic emigrants (RTE) and are markers of thymic output, while the ratio of the later one (sj) by the earliest one (dβ) reflects intrathymic proliferation of thymocytes (18). Measurement of TREC content in splenocytes of *Ghrh*KO mice reveals a twofold increase number of sjTREC compared with age-matched WT mice and a threefold and fourfold increase in dβTREC content at 3 and 6 months, respectively (Figure 1D). Conversely, the intrathymic proliferation estimated by the sj/dβ ratio is reduced in 3-month-old mutant mice. Results were similar in blood PBMC (Figure S1 in Supplementary Material). Collectively, those results indicate an increased thymic output of naïve T cells with decreased intrathymic proliferation. Moreover, TREC analysis shows that thymopoiesis is not impaired at 6 months, with values similar to 3-month-old mice in both WT and mutant (Figure 1D). Unfortunately, we were unable to measure TREC in 18-month-old mice due to the difficulty to obtain such old animals. However, data could be inferred from GH supplementation experiment, where TREC are measured in blood of 3- and 18-month-old mice 2 weeks before GH treatment and show a clear reduction of thymic output at 18 months in both strains while intrathymic proliferation is reduced only in WT mice (Figure 3E; Figure S1 in Supplementary Material). This reveals that a decline in thymopoiesis occurs similarly in WT C57BL/6 and *Ghrh*KO between 6 and 18 months of age.

### Lymphocytes Distribution in Periphery Is Slightly Disturbed in GhrhKO Mice

**Figure 2:**
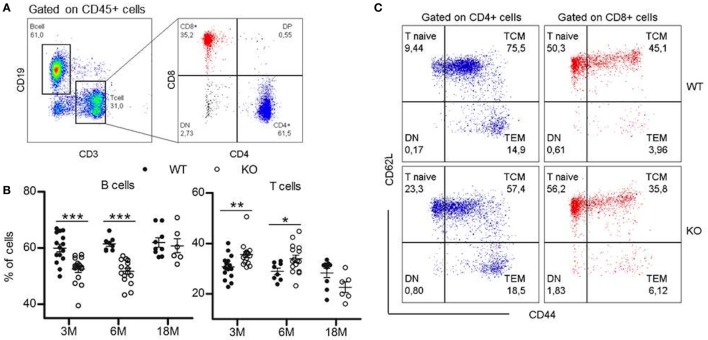
Lymphocytes distribution in periphery. **(A)** Representative flow cytometry plots showing immunophenotyping of peripheral lymphoid compartments (blood, spleen, and lymph nodes). CD19^+^ B cells and CD3^+^ T cells are analyzed within the CD45^+^ population. T cell population is divided into CD4^+^ and CD8^+^ T cells. 20,000 CD45^+^ events are recorded. **(B)** Proportions of B and T cells in the spleen of KO (white circles, *n* = 6–16) compared with wild-type (WT) (black circles, *n* = 8–15) mice at 3, 6, and 18 months. Data (mean ± SEM) are representative of two to three independent experiments except for the KO18M group analyzed in a single experiment. Unpaired *t*-test was used for statistical analysis. ***p < 0.001, *p < 0.05. **(C)** Representative flow cytometry plots for phenotyping of naïve and memory T cells in the spleen of 3-month-old WT and KO mice. Naïve (CD44^low^CD62L^hi^), TCM (CD44^hi^CD62L^hi^), and TEM (CD44^hi^CD62^low^) cells are analyzed within the CD3^+^CD4^+^ (blue) or CD3^+^CD8^+^ (red) populations.

To determine if somatotrope deficiency could also affect peripheral lymphocytes, flow cytometry analyses of spleen, inguinal LNs, and blood were performed. *Ghrh*KO mice present an approximately 10% reduction of B-cell frequency at 3 and 6 months, while T-cell proportion is increased (Figure 2A,B; Table 3). Differences tend to attenuate with aging, since 18-month-old KO mice are not different from age-matched WT mice. Among T cells, distribution of CD4 and CD8 T cells is also disturbed in mutant mice, but depends on age and organ analyzed. Indeed, there is no difference in the spleen of 3-month-old mutant mice. At 6 months, the slightly decreased proportion of CD4 T cells and the increased proportion of CD8 T cells in *Ghrh*KO observed when comparing by *t*-test is not significant when two-way ANOVA was used (Table 3; Table S3 in Supplementary Material) suggesting the time variation biased the difference between mutant and WT mice. On the contrary, the increased proportion of CD4 and the decrease in CD8 is observed at 18 months whatever the test used (Figure 2B; Table 3; Table S3 in Supplementary Material). In LN, mutant mice shows a constant increased in CD4 T cells and decreased in CD8 T-cell frequencies (about 5%) compared with normal mice (Table 3; Table S3 in Supplementary Material). The CD4/CD8 ratio in LN and blood of both normal and mutant mice decreases with time, an expected effect of aging (22).

**Table 3:** Effects of somatotrope deficiency on frequency of lymphocytes subpopulations in spleen, lymph node (LN), and blood.

	3 Months		6 Months		18 Months	
			
Frequency (% of parent population)	C57BL/6 wild-type (WT) (*n* = 15)^c^	*Ghrh*KO (*n* = 16)^c^	C57BL/6 WT (*n* = 8)^b^	*Ghrh*KO (*n* = 16)^c^	C57BL/6 WT (*n* = 9)^c^	*Ghrh*KO (*n* = 6)^a^
**Spleen**						
B cell	60.0 ± 1.36	52.5 ± 1.18***	61.4 ± 0.85	51.7 ± 1.06***	61.9 ± 1.67	60.8 ± 2.52
T cell	30.6 ± 1.23	35.5 ± 1.17**	28.9 ± 1.25	33.9 ± 1.41*	28.3 ± 1.84	22.6 ± 2.12*
T CD4	57.4 ± 0.66	56.2 ± 1.04	57.0 ± 0.40	54.3 ± 0.86*	55.7 ± 1.08	62.9 ± 2.52*
T CD8	36.7 ± 0.61	36.9 ± 1.08	36.0 ± 0.67	38.8 ± 0.81*	37.1 ± 0.93	31.4 ± 2.83*
CD4 naive	12.0 ± 0.62	29.7 ± 1.30***	12.1 ± 0.75	21.0 ± 1.36***	3.4 ± 0.36	9.6 ± 2.67
CD4 TCM	61.7 ± 2.20	45.6 ± 1.42***	52.1 ± 2.03	50.7 ± 1.39	21.2 ± 2.57	21.5 ± 4.04
CD4 TEM	24.6 ± 1.62	23.7 ± 1.47	35.5 ± 2.08	27.9 ± 1.99*	74.7 ± 2.60	68.5 ± 6.33
CD8 naive	47.9 ± 1.05	57.5 ± 1.26***	47.1 ± 1.74	59.2 ± 1.55***	17.9 ± 2.18	29.8 ± 6.02
CD8 TCM	40.8 ± 1.31	31.0 ± 1.02***	42.6 ± 1.57	31.2 ± 1.48***	59.4 ± 3.69	44.5 ± 2.15**
CD8 TEM	7.1 ± 0.69	7.3 ± 0.59	9.4 ± 0.57	7.6 ± 0.42*	22.4 ± 2.89	24.4 ± 4.17
Treg	14.0 ± 0.38	17.3 ± 0.60***	17.9 ± 0.69	18.0 ± 0.61	29.3 ± 1.98	29.2 ± 2.43
**LN**						
B cell	33.8 ± 1.85	26.5 ± 1.56**	39.8 ± 3.42	27.6 ± 1.94**	53.6 ± 2.50	48.1 ± 4.08
T cell	63.2 ± 1.82	70.1 ± 1.60**	56.5 ± 3.43	68.2 ± 2.27*	43.3 ± 2.66	42.3 ± 4.04
T CD4	53.1 ± 0.71	57.8 ± 1.13**	50.4 ± 0.48	55.5 ± 1.05**	42.0 ± 0.95	48.4 ± 1.99*
T CD8	43.5 ± 0.74	39.0 ± 1.15**	44.5 ± 0.38	40.8 ± 1.00*	49.8 ± 1.07	42.4 ± 2.46*
CD4 naive	15.2 ± 0.97	35.7 ± 1.72***	16.6 ± 0.66	31.4 ± 1.78***	10.6 ± 1.33	24.5 ± 3.37**
CD4 TCM	72.1 ± 1.80	48.7 ± 1.95***	63.0 ± 4.68	54.3 ± 1.70*	52.9 ± 2.39	37.6 ± 3.25**
CD4 TEM	11.5 ± 0.99	13.4 ± 1.29	18.7 ± 4.17	13.3 ± 0.88	36.2 ± 3.01	37.1 ± 5.15
CD8 naive	57.6 ± 1.19	66.4 ± 1.21***	59.7 ± 1.31	65.1 ± 1.51	39.5 ± 2.72	40.2 ± 4.64
CD8 TCM	37.3 ± 1.25	25.4 ± 0.84***	34.5 ± 1.44	28.0 ± 1.73	54.1 ± 2.21	47.5 ± 4.29
CD8 TEM	2.6 ± 0.21	2.8 ± 0.21	3.5 ± 0.27	3.0 ± 0.25	5.9 ± 0.81	9.2 ± 1.66
Treg	12.5 ± 0.53	12.5 ± 0.22	16.1 ± 0.51	16.3 ± 0.70	30.3 ± 2.13	30.7 ± 2.12
**Blood**						
B cell	46.1 ± 2.65	43.3 ± 2.55	54.2 ± 3.10	43.1 ± 5.39	75.7 ± 3.58	51.2 ± 5.26**
T cell	38.7 ± 1.64	30.4 ± 3.62*	29.8 ± 3.83	33.3 ± 6.31	17.2 ± 2.00	26.5 ± 3.78*
T CD4	54.9 ± 1.55	55.3 ± 0.96	52.2 ± 1.94	49.6 ± 3.53	35.5 ± 2.03	51.5 ± 3.38**
T CD8	42.5 ± 1.58	37.3 ± 1.43*	44.9 ± 2.09	46.1 ± 2.95	58.2 ± 2.53	44.5 ± 3.79*
CD4 naive	15.8 ± 0.88	27.1 ± 4.20**	19.3 ± 1.93	31.9 ± 2.87**	18.7 ± 6.06	30.6 ± 4.45
CD4 TCM	76.7 ± 1.72	36.6 ± 5.31***	67.3 ± 2.22	49.9 ± 4.06**	51.9 ± 5.02	40.9 ± 4.03
CD4 TEM	6.6 ± 0.90	27.8 ± 7.45***	12.1 ± 1.79	14.0 ± 2.83	34.9 ± 6.08	28.3 ± 8.25
CD8 naive	53.4 ± 2.86	31.7 ± 6.19**	48.6 ± 3.18	44.9 ± 4.03	32.5 ± 5.99	38.8 ± 6.74
CD8 TCM	42.6 ± 3.00	42.8 ± 1.87	40.5 ± 2.03	41.52 ± 2.39	49.0 ± 5.05	50.5 ± 4.66
CD8 TEM	2.6 ± 0.46	17.4 ± 5.08*	8.4 ± 1.81	8.34 ± 1.71	20.8 ± 6.55	10.5 ± 2.94
Treg	9.2 ± 0.98	8.0 ± 0.63	7.5 ± 0.90	7.8 ± 0.44	13.0 ± 0.92	11.2 ± 1.99

*Data (mean ± SEM) are representative of one^a^, two,^b^ or three^c^ independent experiments*.

Values significantly different from age-matched WT mice ***p < 0.001, **p < 0.01, *p < 0.05.

*CD19^+^ B cells and CD3^+^ T cells are analyzed within the CD45^+^ population. T cell population is divided into CD4^+^ and CD8^+^ T cells. 20,000 CD45^+^ events are recorded. Naïve (CD44^low^CD62L^hi^), TCM (CD44^hi^CD62L^hi^), and TEM (CD44^hi^CD62^low^) cells are analyzed within de CD3^+^CD4^+^ or CD3^+^CD8^+^ populations. FoxP3^+^ Treg cells are studied inside the CD4^+^ SP population*.

Differences in lymphocytes subsets distribution were further studied by exploring the naive or memory character of CD4 and CD8 T cells. In mice, the low expression of CD44 is specific to naive T cell, while differential expression of L-selectin CD62L allows to separate central memory T cells (TCM) and effector memory T cells (TEM) in the CD44^high^ quadrant. The pool of naive T cells (CD44^low^CD62L^hi^) is greater in *Ghrh*KO compared with WT mice, with an doubled frequency of naive cells inside the CD4 subset and a moderate increase of 10% among CD8 T cells (Figure 2C; Table 3). On the contrary, the proportion of memory cells is reduced, mostly through the CD44^hi^CD62L^hi^ central memory (TCM) pool. This is consistent with the observed increase of TREC in *Ghrh*KO mice, since TREC are present in newly formed naive cells exported from the thymus.

Finally, since Treg proportion seems higher in thymus of *Ghrh*KO mice, FoxP3^+^ Treg cells were next analyzed among CD4 T cells in periphery. As expected, Treg compartment enlarges with time, but in similar proportions between *Ghrh*KO and WT mice, except in the spleen of 3-month-old *Ghrh*KO mice where Treg proportion is higher than in control mice (*p* < 0.001; Table 3; Table S3 in Supplementary Material). Taken together, those data demonstrated changes in the distribution of some lymphocytes subsets in peripheral lymphoid organs, without any evidence of lymphopenia.

### GH Supplementation Is Unable to Restore Normal Phenotype in GhrhKO Mice

**Figure 3:**
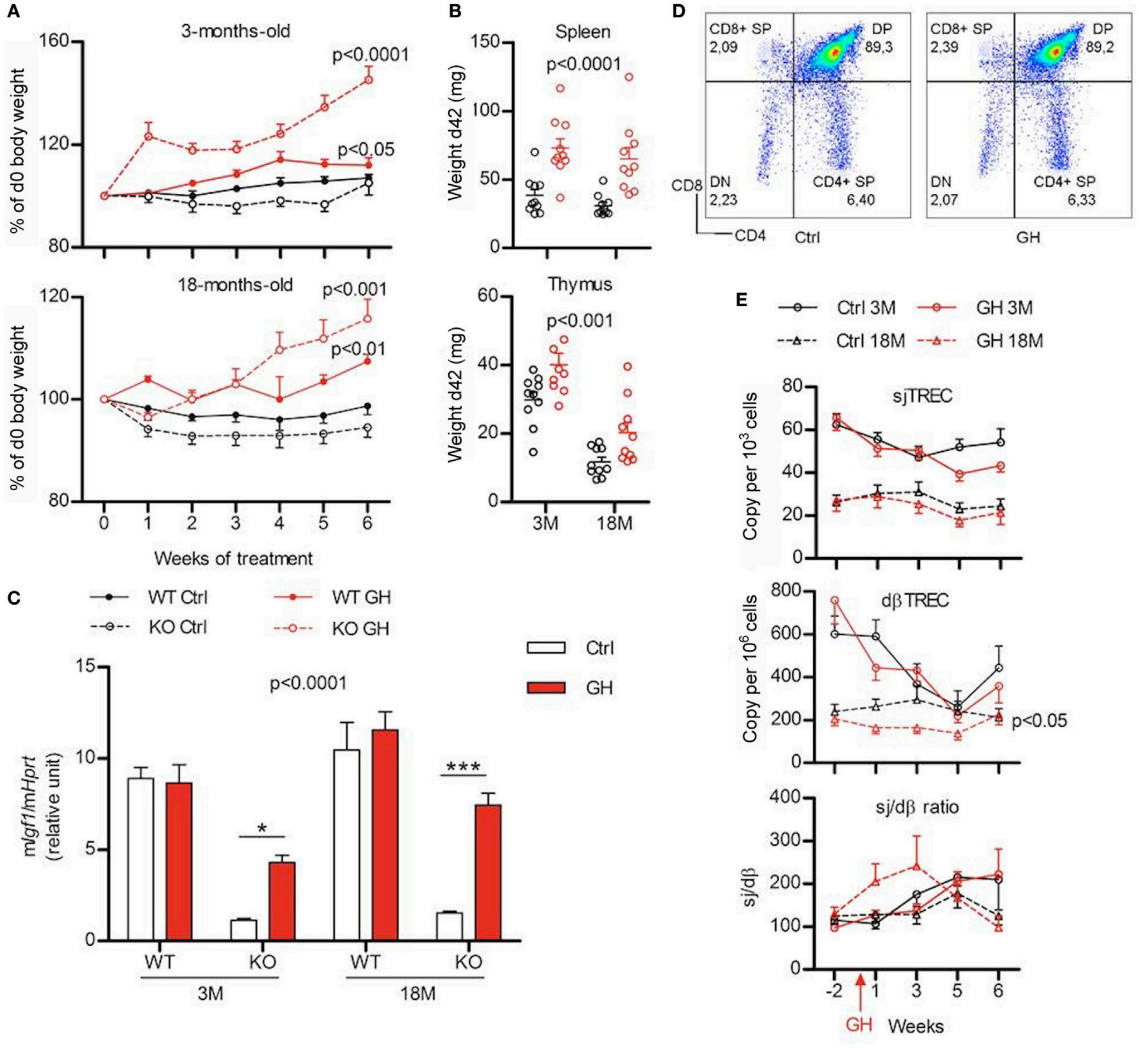
Effects of growth hormone (GH) supplementation (6 weeks) on metabolic and immune parameters. **(A)** Weight variations expressed in percentage of starting value (weight at d0) are shown for 3- (above) and 18-month (below) old wild-type (WT) (filled line) and KO mice (dotted line) injected with GH (red) or control Dulbecco’s phosphate-buffered saline (black). Two-way ANOVA test (time and treatment) was used for statistical analyses (*n* = 7–10 per group). **(B)** Igf1 expression in the liver of KO and WT mice after 6 weeks of GH (red) or control (white) treatment was measured by real-time quantitative PCR and normalized to expression of Hprt. Two-way ANOVA test (treatment and strain) with Bonferroni post-test were used for statistical analyses (*n* = 6–10 per group). ***p < 0.001, *p < 0.05. **(C)** Absolute weight of spleen (above) and thymus (below) of young and old KO mice after 6 weeks of GH (red) or control (black) treatment. Two-way ANOVA test (treatment and age) was used for statistical analyses (*n* = 10 per group). **(D)** Representative flow cytometry plots of immunophenotyping of thymus from control- and GH-injected mouse. **(E)** Variations of peripheral sjTREC, dβTREC, and sj/dβ ratio during GH supplementation (red) and control (black) in 3- (filled line) and 18-month-old (dotted line) KO mice. Two-way ANOVA test (time and treatment) was used for statistical analyses (*n* = 10 per group). **(A,B,C,E)** Data (mean ± SEM) are representative of two independent experiments.

To determine if GH supplementation could restore a normal phenotype in *Ghrh*KO mice, animals were injected with a replacement dose of human recombinant GH or DPBS as control. Young and old mutant and WT mice were tested to see if GH action varies with aging. The efficiency of GH treatment was validated by its effects on weight and liver IGF1 stimulation (Figures 3A,C). Indeed, both *Ghrh*KO and WT mice show significant weight gain during GH treatment, compared with control mice, even though the effect is far more important (Bonferroni test following two-way ANOVA: *p* < 0.001 KO vs WT for 3 months mice after 6 weeks of GH treatment) in KO mice with a ~45% increase for 3 months compared with ~11 for WT mice (Figure 3A). Spleen and thymus in mutant mice are also significantly heavier in GH-treated relatively to DPBS-injected, particularly the spleen whose mass is doubled compared with control-injected mice (Figure 3B). Moreover, GH treatment stimulates *Igf1* expression in the liver of *Ghrh*KO mice, although it does not reach values in normal mice (Figure 3C). This demonstrates that a 6-week treatment with GH is able to compensate the somatotrope deficiency by inducing weight gain and IGF-1 stimulation without completely restoring values measured in normal mice.

The impact of GH treatment upon immune system was first evaluated in the thymus. No differences are observed between GH- or control-injected mice regarding the distribution of the four thymocyte sub-population and Treg cells in the thymus (Figure 3D). Similarly, TREC number and Sj/dβ ratio during GH treatment does not significantly differ from control-injected mice (Figure 3E). Young animals exhibit a decrease in both Sj and dβTREC during treatment with GH or vehicle alone that does not appear in old mice. Nevertheless, no differences appear at 3 or 18 months neither between control and GH-injected animals. This suggests that aging is not a factor that sensitizes mice to GH.

Next, the ability of GH injections to restore lymphocytes phenotype in periphery was also studied. There were no detectable differences in the frequency of all lymphocytes subtypes in the LN of GH-treated mutant mice compared with control (Figure S2 in Supplementary Material, lower panel). However, in the spleen, GH significantly increased B-cell frequency and decreased T cells (*p* = 0.002 and 0.001, respectively; Figure S2 in Supplementary Material, upper panel), increased frequency of CD4 T cells (*p* = 0.043), as well as increased CD8 naive pool and decreased CD8 TCM (*p* = 0.025 and 0.005, respectively, data not shown). The other subtypes were not affected by GH treatment. Finally, a blood sample was taken 1 week of two to follow the frequency of lymphocytes across the time. Flow cytometry experiments revealed huge week-to-week variations in all the groups studied (Figure S2 in Supplementary Material). Even if two-way ANOVA analysis revealed some significant effects of the treatment (decreased B cells, increased T cells, and decreased CD8 TCM in KO3M; increased CD4 TEM and decreased CD8 naïve T cells in KO18M, data not shown), it is still unclear if the differences are due to a real effect of GH or to random variations that would affect both control and GH-treated mice. Globally, those results suggest that GH supplementation is not sufficient to restore a normal phenotype in *Ghrh*KO mice.

### GhrhKO Mice Show a Delayed Recovery of Their Thymic Volume After DXM-Induced Thymic Atrophy

**Figure 4:**
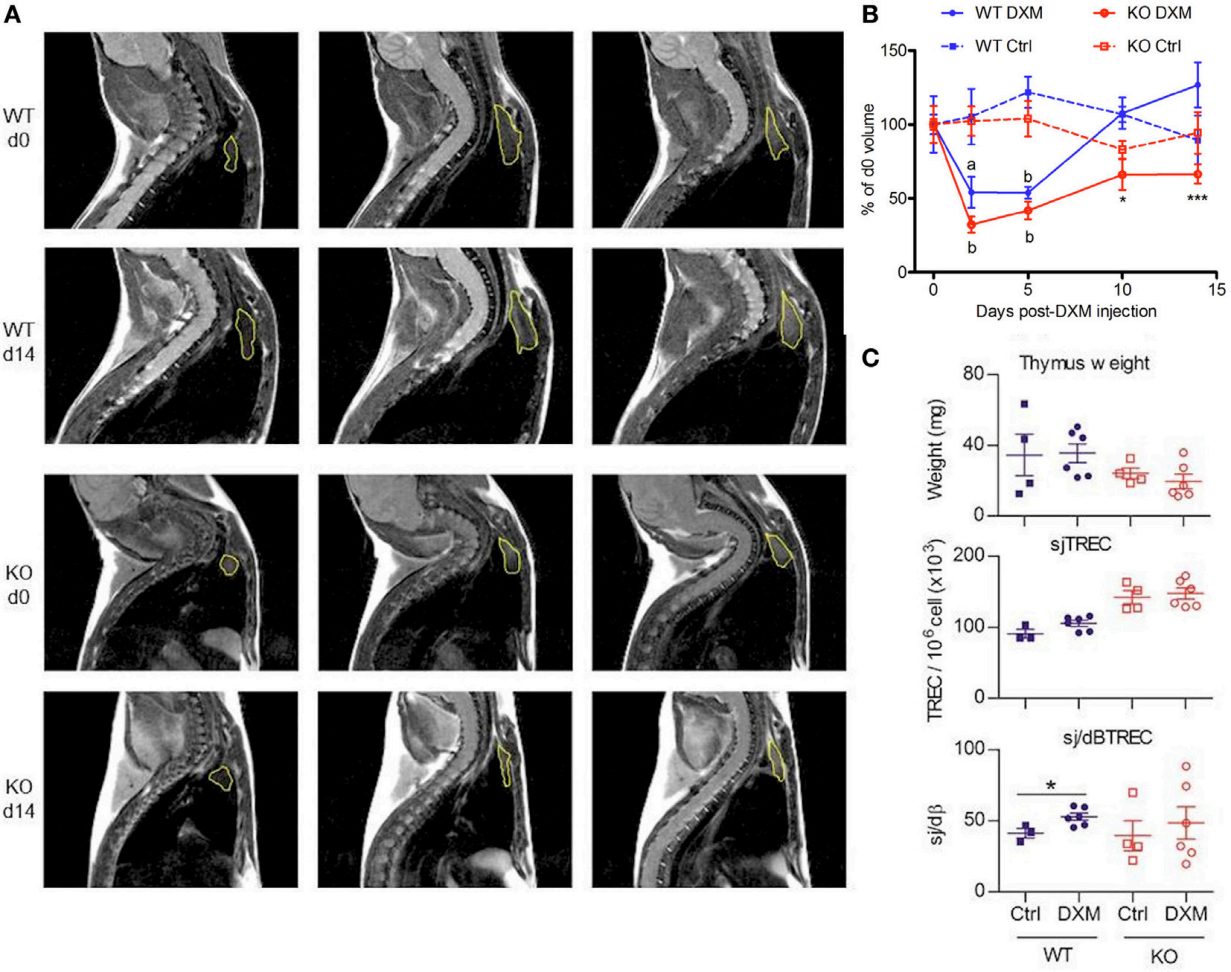
Follow-up of dexamethasone (DXM)-induced atrophy and recovery. **(A)** Representatives MRI slices showing thymus [region-of-interest (ROI), yellow] of wild-type (WT) and KO at d0 and d14 after DXM injection. Lines show three successive slices around the maximal ROI. **(B)** Evolution of thymic volumes normalized in% of d0 value is shown for WT (blue) and KO (red) mice treated with DXM (filled line, *n* = 6 per group) or control solution (dotted line, *n* = 4 per group). Paired *t*-test was used for effect of DXM vs d0. ^a^*p*: < 0.05, ^b^*p* < 0.01. Two-way ANOVA test (time and treatment) with Bonferroni post-test (WT vs KO for DXM or Ctrl treatment) was used for statistical analyze of genotype variation. *p < 0.05, ***p < 0.001. **(C)** Thymus weight, sjTREC and sj/dβ ratio of WT (blue) and KO (red) mice at d15 post DXM- (circle, *n* = 6 per group) or control-injection (square, *n* = 4 per group). Unpaired *t*-test was used for statistical analysis. *p < 0.05. **(B,C)** Data (mean ± SEM) are representative of two independent experiments.

Stress hypothesis suggests that somatotrope deficiency could lead to inability for the immune system to correctly respond to stressful events. To explore this hypothesis, *Ghrh*KO mice were challenged with DXM, a synthetic GC that induces a reversible thymic atrophy. After a single DXM injection, MRI sessions were performed at days 0, 2, 5, 10, and 14 to quantify thymic volumes in a longitudinal follow-up (Figure 4). Both WT and KO mice showed a significant loss of more than 50% of thymic volume at day 2, demonstrating the DXM-induced atrophy. At day 5, thymic volume increased to reach normal values as soon as day 10 in WT mice and even rise above starting volume at day 14. Even if statistical significance was low (*Ghrh*KO d10 vs *Ghrh*KO d0: *p* = 0.616, *Ghrh*KO d14 vs *Ghrh*KO d0: *p* = 0.505, paired *t*-test), *Ghrh*KO DXM-injected mice did not seem to completely restore their thymic volumes at days 10 and 15 (only 70% of the starting volume) but volumes were not significantly different from control-injected group, probably because of high variation rate in control mice. However, the recovery (assessed in percentage of corresponding starting value for each mouse) was significantly different between WT and KO mice at days 10 and 14 (Figure 4B). This suggests a delayed restoration of thymic volumes after DXM-induced thymic atrophy in somatotrope-deficient mice compared with WT mice. However, this difference was not supported by analysis of thymus weight and cellularity obtained after sacrificing the mice at day 15 (Figure 4C). Indeed, they were no difference between control and DXM-injected animals in both WT and KO mice regarding those parameters, neither for TREC intrathymic content. Nevertheless, intrathymic proliferation reflected by sj/dβ ratio was higher in DXM-injected compared with control for WT but not mutant animals (Figure 4C). This revealed an increased thymic activity in WT mice 2 weeks after DXM injection, which was not observed in *Ghrh*KO mice. Altogether, MRI analyses of thymic volumes and sj/dβ ratio results suggested a delayed and less efficient thymic recovery in *Ghrh*KO mice after DXM-induced thymic atrophy while weight and cell number measures showed normal thymic restoration after 15 days. This apparent discrepancy might be attributed to the individual variation bias that cannot be avoided when animals are sacrificed at each time point for organ weighing.

## Discussion

Despite a large amount of literature about the effects of GH upon immune system, its implication in immune physiology is still unclear and controversial. Most of the previous works were done in mouse model with multiple pituitary deficiencies (GH, PRL, and thyrotropic hormones), making it difficult to identify the precise role of each hormone. Here, we investigated a model never used for immune characterization with a unique specific deficiency of the somatotropic GHRH–GH–IGF1 axis due to *Ghrh* deletion, in an attempt to elucidate the physiological role of somatotropic hormones in immune system development and function. We found no severe thymic or immunological defect in those mice. Within the thymus, they present a slight reduction in the proportion of the most immature thymocyte subset, but the relative weight and cellularity of this primary immune organ is similar to that in normal mice, even in old animal.

Age-induced changes in lymphocytes distribution in peripheral lymphoid organs were studied in mutant and *Ghrh*^−/−^ mice. It is important to note that we cannot exclude some bias (1 of 20 parameters will reach a *p* < 0.05 randomly, cohort effects in aged animals, etc.) in analysis and interpretation of so many parameters. Therefore, careful and critical interpretation should be applied. Here, only consistent repeated results were taken into consideration. A first conclusion drawn from analysis of spleen, LNs and blood was that the differences observed between normal and *Ghrh*^−/−^ mice at 3 months (i.e., lower B-cell and higher T-cell frequencies and higher proportion of naïve T cells and diminution of memory pool in KO vs WT mice) were maintained throughout life. Altogether, analysis did not reveal a strong differential effect of aging on peripheral lymphocytes between *Ghrh*^−/−^ and normal mice. Both maintained relatively constant proportion of B and T cells and, as expected, they experienced a shift in the pool of naïve to memory T cells, within which mostly TEM were increased. Frequency of CD4 T cells decreased in the blood and LN of normal and mutant mice, but an inverted increase of CD8 frequency was observed only in WT organs. This resulted in a decreased CD4/CD8 ratio in the two compartments of the two types of aged mice, although the intensity of this decrease was more important in WT mice.

In periphery, *Ghrh*KO mice exhibit a decreased frequency of B cells, concomitant with a rise in proportion of T cells. The spleen is the only immune organ that remains smaller than in the WT counterpart when taking into account the body weight of the animal. The pool of naïve T cells is more important in somatotrope-deficient mice, a result also supported by the higher number of TREC in spleen and blood of *Ghrh*KO mice. A 6-week GH supplementation is unable to restore those parameters to normal in our experimental model. Nevertheless, *Ghrh*KO mice do not present any obvious immunodeficiency or thymic atrophy. Taking together, those results indicate that the integrity of the somatotrope axis is not required for T cell immune system development in basal conditions.

Our results are in contradiction with previous work conducted in multi-deficient dwarf mouse model (Snell–Bagg and Ames) where were observed lymphopenia, decreased relative weight thymus, early thymic involution and reduced primary immune response compared to non-dwarf animals (2–4). This immunodeficiency is characterized by decreased number of thymic cells and dramatic decreased in proportion of DP thymocytes (23). Moreover, GH treatment could partially restore immune parameters (3, 23). Therefore, authors concluded that GH had significant effect on T cell development within the thymus. However, in most of the studies demonstrating a decreased thymic cellularity, the absolute number of cells was compared without normalizing to the smaller size of dwarf mice (23), and it cannot be so assumed that the smaller thymus size is due to a direct effect of GH upon the thymus or to spatial pressure linked to growth retardation. We and others (10, 11) demonstrated that even though a diminished absolute number of cells, GH-deficient mice had a normal thymic cellularity when corrected to their smaller size.

Other works are in agreement with our findings in *Ghrh*KO mice. The B cell frequency decrease is coherent with the previously observed impairment of bone marrow B cell production in dwarf mice model (11, 24). Very interestingly, this specific absolute and relative decrease in spleen size has been observed in human with a GHRH receptor mutation leading to dwarfism, establishing a link between our animal model and observation in human (25). A team studied dwarf Snell–Bagg mice and found no differences in lymphocytes distribution or function in thymus, while the spleen demonstrated a higher frequency of T lymphocytes and lower frequency of B lymphocytes compared with control (10). The number of splenic cells—but not thymic lymphocytes—was 50% of normal counterparts when corrected to total body weight, similar to what we found in *Ghrh*KO mice. Another group, using a panel of mouse strains affected with different pituitary hormone deficiencies, showed that primary B cell development defect was not dependent on hypophysial hormones, but was controlled by thyroid hormones. Nevertheless, GH could be involved in B cell reduction within secondary lymphoid organs (11). Indeed, thyroid axis-deficient mice exhibit a defect in bone marrow B lymphopoiesis and normal splenic B cells frequency, while the opposite was found in GH-deficient *lit/lit* mice. In both cases, thymus was unaffected by hormone deficiency. Our study in *Ghrh*KO mice confirms these observations, since we observed an almost normal thymus, but diminished B cells frequency in periphery (spleen, LNs, and blood). This reduction goes along with increasing proportion of T cells. Interestingly, the growing literature about effects of pituitary hormones upon B-lymphopoiesis strongly suggests a role for GH and IGF-1 as positive regulators of B cells (11, 26–29). It has been shown that GH receptor has a wider expression on B cells that on T cells (50% compared with 20%, respectively) (30), suggesting a higher sensitivity to GH for this lymphocyte subset. Moreover, there are evidences that IGF-1 is able to increase the amount of bone marrow B lineages cells and splenic B cells as well as accelerate B cell reconstitution after bone marrow transplantation (26, 31). Similarly, GH-transgenic mice exhibits higher number of total lymphocytes, an effect more important in B cells than T cells (28). Taking this into account, it is not surprising that *Ghrh*KO and *lit/lit* mice display B lymphopenia.

One surprising result is the marked increase in the number of TREC in *Ghrh*KO mice, with an opposite decrease of sj/DJβTREC ratio. Previous works from our lab and others are in favor of a positive role of somatotrope hormones upon TREC production. In GH-deficient patient, withdraw of GH treatment induced a drop in sjTREC frequency and sj/DJβTREC ratio, followed by recovery after GH resumption (9). Furthermore, HIV+ patient treated with GH showed increased TREC frequency in PBMC (8). In mice, IGF-1 administration resulted in significant increase in TREC number measured in thymus and periphery (32). Therefore, we expected that GH and IGF-1 deficiencies could lead to TREC diminution measured in blood. One hypothesis to explain our opposite result here is that GH absence affects peripheral proliferation of cells and/or cell activation in response to antigen more than thymic proliferation. Indeed, TREC, which are excision circles of DNA resulting from T cell receptor rearrangement, are stable in the cell but not duplicated during mitosis, leading to their progressive dilution across peripheral proliferation. So, interpreting sjTREC content as a marker of thymic output should be done carefully, regarding this dilution bias. On the contrary, the intrathymic proliferation rate estimated by sj/dβTREC ratio is independent of peripheral proliferation since it represents the ratio between a TREC created lately in the thymus (sj) to one formed early (dβ). Here, the apparent increase in thymopoiesis indicating by the higher number of TREC could be a false interpretation due to a less important proliferation rate in periphery of *Ghrh*KO mice. *In vitro* and *in vivo* studies demonstrated that GH and IGF-1 are able to stimulate T cell proliferation (32, 33). Importantly, sj/dβTREC ratio could truly reflect a decreased intrathymic proliferation in young mutant mice, as expected according to studies described above. A second hypothesis would involve a decreased cell activation and is reinforced by the high frequency of naïve T cells found in somatotrope-deficient mice. If *Ghrh*KO lymphocytes are less sensitive to antigen stimulation, they do not undergo the activation process, which implicates clonal proliferation and induction of memory cells. Therefore, TREC are less diluted, and pool of naïve cells stays more important than in normal mice. This theory of hyposensibility to antigen activation could explain the decreased sensitivity of *Ghrh*KO mice to induction of EAE (34). Moreover, this is in agreement with the current stress hypothesis (13), according to which pituitary hormones are immunoregulators that counteracts negative effects of stress, including physiological and biological stress, like antigen challenge. The absence of one or more of those stress hormones could result in inability of the immune system to deal with stressful situations.

The stress hypothesis was first proposed by Dorshkind and Horseman based on their observation that mice deficient for GH/IGF-1, PRL, or thyroid hormones have a normal humoral and cellular response (35), a result in contradiction with previous statements in literature (2, 4, 23, 36, 37). Afterward, they discovered that *Snell–Bagg* dwarf mice housed in non-stressful conditions, separately from their normal littermates, had no thymopoiesis defects, in contrast to animals held in less stringent conditions (13, 38). Reviewing literature in this context reconciles the contradictory findings about immunodeficiency in pituitary-deficient mice. Most of the studies showing a depressed immune system dependent on pituitary hormones (2–4) were conducted 40 years ago when housing conditions were less healthy and could be source of physiological and psychological stress. On the opposite, we and others (10, 38), keeping the animals in stress-limited and highly sanitary environment, found normal thymus and immune system. This hypothesis is reinforced by the evidence that GH can inhibit cortisol-induced lymphopenia in hypophysectomized rats (39). Moreover, GH secretion is stimulated after stress exposition (40). A mechanism for this GH inhibition of GCs action involves the Jak2/Stat5 pathway, one of the GH-signaling pathways (41). It has been shown that Stat5 protein can form a complex with the GC receptor which diminishes the activation of promoters containing GC response elements and therefore inhibits GC-induced gene activation (42). PRL, another pituitary hormone which share the Stat5 signaling pathway, has been shown to suppress *in vivo* lymphocytes apoptosis induced by DXM, a synthetic GC (43). The role of PRL as anti-stress hormone was confirmed in mouse experimental *Trypanosoma cruzi* infection, characterized by increased levels of GC, and inversely decreased levels of PRL, and where PRL restoration limited thymic atrophy and DP thymocytes apoptosis (44). It is plausible that GH, sharing the transduction pathway with PRL, could act through a similar mechanism on stress-induced immunosuppression. The presently described *Ghrh*KO mouse constitutes an interesting experimental model to assess this question. According to the stress hypothesis, somatotrope deficiency in those mice drives an altered resistance to stress. Indeed, mimicking GC-induced stress by DXM administration reveals here a slower thymus recovery in mutant mice, as demonstrated by MRI quantification of thymic volumes and sj/dβ ratio. To the best of our knowledge, this is the first time thymus regeneration after DXM-induced atrophy is longitudinally followed by MRI. This method was validated by Brooks and colleagues as a non-invasive way to measure thymus involution induced by DXM, giving high statistical power using less animals compared with measurement of tissue weight (35). However, considering the high variability observed in thymic volumes of control-injected mice and the small size of each group, results should be interpreted with caution. Moreover, thymus weight and cellularity as well as TREC number measured 1 day after the last MRI session showed normal values in mutant mice injected with DXM. Altogether, those results do not allow to firmly validate the stress hypothesis. Challenges with other type of stress, like infectious stress, are currently performed.

Aging is considered as a stressful situation for the immune system. It is well known that thymus undergo severe atrophy with aging, and elderly are less resistant to infections and autoimmune diseases (45). GH deficiency has been described to extend lifespan and delay immune aging (46, 47). For example, a study in Snell–Bagg and GHRH-R-deficient mice showed a 40% increased longevity regarded to WT mice and some parameters of aging immune system were also improved: similar proportion of memory cells and T cell function compared with young animals (47). This is consistent with the antagonistic pleiotropy theory, according to which genes conferring reproductive advantages are selected throughout evolution, despite their deleterious effects at long term (48). However, in our study, the somatotrope deficiency is not an aggravating factor for the aging immune system. Thymus atrophy, seen by the decreased weight and cellularity of the organ as well as TREC number, is parallel between mutant and normal mice. It should be pointed that our model is a genetic defect in GHRH that affect all the somatotrope axis since the beginning of development. The results should therefore be taken with precautions when comparing to acquired GH deficiency like it is postulated with aging.

Another surprising conclusion of this work is the inefficiency of GH supplementation to restore immune parameters, despite the clear metabolic effects of the treatment. Indeed, GH-daily injection in *Ghrh*KO mice results in increased body, spleen, and thymus weight and stimulation of IGF-1 production in the liver, as expected (16). However, none of the immune parameters analyzed, i.e., thymic and peripheral lymphocytes phenotype and TREC content, was modified by the 6-week-long treatment, even in old animals. This is surprising since numerous works showed that GH injection had beneficial effects on thymic function (3, 23), especially in aged rodents where it could reverse thymic involution (5) and on antibody production (37). Once again, stress hypothesis can explain the discrepancy between our results and literature. Another possibility is that IGF-1 is the main actor of somatotrope actions in the immune system (9, 49), and the dose of GH injected (1 mg/kg) was not able to induce the production of a sufficient amount of IGF-1. Indeed, IGF-1 was under detection limit of 4.0 ng/ml in serum of GH-injected *Ghrh*KO mice (data not shown).

Altogether, these data show that the severe somatotrope deficiency of *Ghrh*^−/−^ mice essentially impacts the spleen and B compartment of the adaptive immune system, while it only marginally affects thymic function and T cell development. Our laboratory is now investigating the susceptibility of *Ghrh*^−/−^ mice to T-independent and T-dependent pathogens.

## Ethics Statement

This study was carried out in accordance with the European recommendations for animal health care and the protocols were approved by the Animal Ethics Committee of the University of Liege GIGA Institute.

## Author Contributions

GB and KF are equal first authors and performed all experiments. CR-C assisted technically GB and KF. GBe and AP performed MRI analyses. RS provided the laboratory with GHRH-KO mice. GB, GBe, AP, HM, and VG designed the protocol of experiments. GB, VG, and HM wrote the manuscript. VG and HM are equal last authors and supervised the whole experimental work.

## Conflict of Interest Statement

The authors declare that the research was conducted in the absence of any commercial or financial relationships that could be construed as a potential conflict of interest.

## Abbreviations

LN, lymph node; WT, wild-type; FSC, forward scatter; SSC, side scatter; TREC, T-cell receptor excision circle; Sj, signal joint; RT-qPCR, real-time quantitative PCR; DN, double-negative; DP, double-positive; SP, single-positive; TCM, central memory T cell; TEM, effector memory T cell; GC, glucocorticoid.
