# Supplementary material for: The Severe Deficiency of the Somatotrope GH-Releasing Hormone/Growth Hormone/Insulin-Like Growth Factor 1 Axis of Ghrh−/− Mice Is Associated With an Important Splenic Atrophy and Relative B Lymphopenia
Source: Front Endocrinol (Lausanne). 2018 Jun 6;9:296. doi: 10.3389/fendo.2018.00296 (PMC5997896; doi:10.3389/fendo.2018.00296)
Supplement: Supplementary file 1 [file image_1.PDF]

Supplemental data

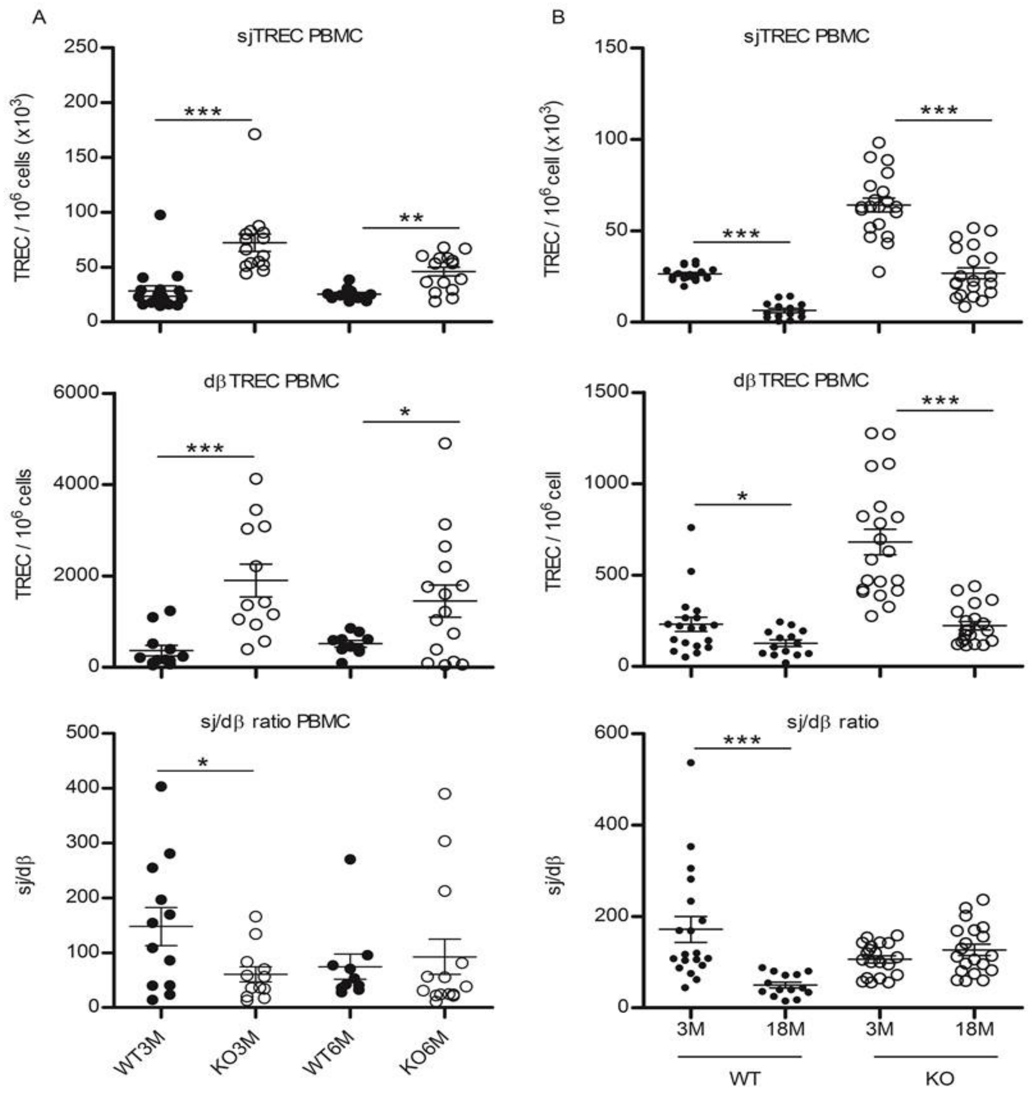

Figure S1: TREC quantification in PBMC and comparison between young and old mice. (A, B) sjTREC, dβ TREC and sj/dβ ratio in (A) PBMC of KO (○, *n* = 15-16) and WT (●, *n* = 12-17) mice at 3 and 6 mo and in (B) PBMC of KO (○, *n* = 14-20) and WT (●, *n* = 14-19) mice of 3 and 18 mo two weeks before GH treatment (already presented in Fig 3E). Data (mean ± SEM) are representative of 2 or 3 independent experiments. Unpaired t-test or Mann-Whitney test were used for statistical analysis according to the Gaussian distribution of each set of data. *** $p < 0.001$, * $p < 0.05$.

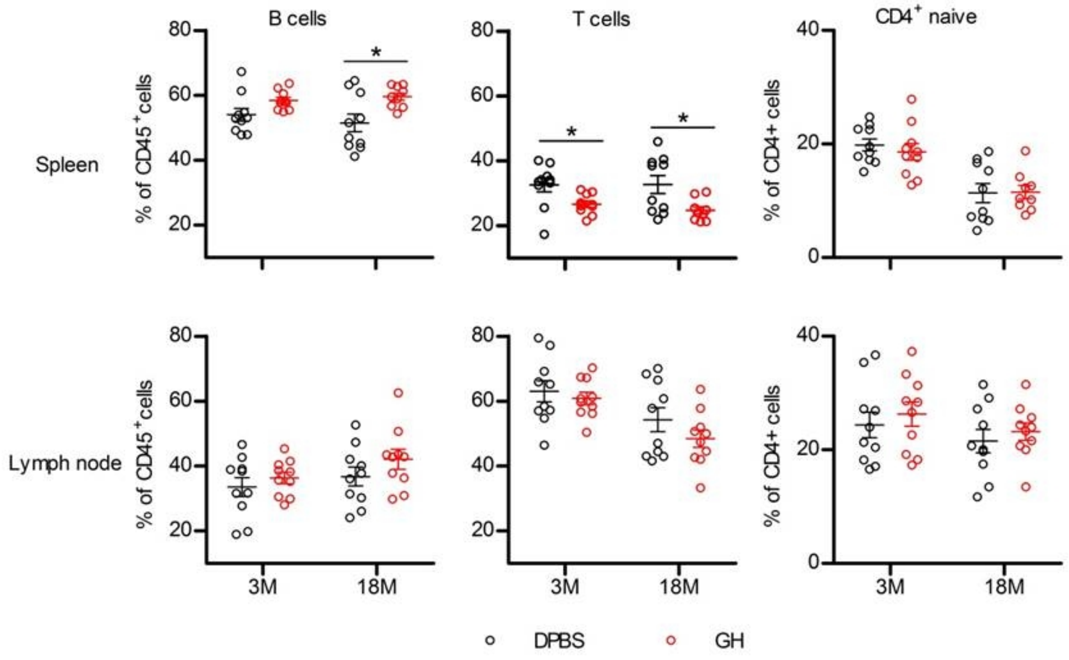

Figure S2: Peripheral lymphocytes distribution in GH-treated *Ghrh*KO mice. Proportion of B and T cells within CD45+ population and naïve cells within CD4+ T cells in spleen and lymph nodes of young (3M, *n*= 10 per group) and old (18M, *n* = 10 per group) KO mice after 6 weeks of GH (red) or control (black) treatment. Unpaired t-test was used for statistical analysis. Data (mean ± SEM) are representative of 2 or 3 independent experiments. * $p < 0.05$.
