# Supplementary material for: The Severe Deficiency of the Somatotrope GH-Releasing Hormone/Growth Hormone/Insulin-Like Growth Factor 1 Axis of Ghrh−/− Mice Is Associated With an Important Splenic Atrophy and Relative B Lymphopenia
Source: Front Endocrinol (Lausanne). 2018 Jun 6;9:296. doi: 10.3389/fendo.2018.00296 (PMC5997896; doi:10.3389/fendo.2018.00296)
Supplement: Supplementary file 2 [file table_1.DOC]

Table I. Two-way ANOVA for effects of somatotrope deficiency on weight and number of cells of lymphoid organs
Data (mean ± SEM) are representative of one^1^, two^2^ or three^3^ independent experiments. ND: not determined.
Bonferroni significant difference between age-matched KO *vs* WT mice : *** *p* < 0.001, ** *p* < 0.01, * *p* < 0.05.
Bonferroni significant difference from 3M mice :(A) *p* < 0.001, (B) *p* < 0.01, (C) *p* < 0.05.
Bonferroni significant difference from 6M mice :(a) *p* < 0.001, (b) *p* < 0.01, (c) *p* < 0.05.

|  | Two-Way ANOVA P value summary | | | 3M | |  | 6M | |  | 18M | |
| --- | --- | --- | --- | --- | --- | --- | --- | --- | --- | --- | --- |
|  | Interaction | Strains | Age | C57BL/6 WT  (*n* = 3♂ 12♀)^3^ | *Ghrh*KO  (*n* = 10♂ 6♀)^3^ |  | C57BL/6 WT  (*n* = 2♂ 6♀)^2^ | *Ghrh*KO  (*n* = 4♂ 12♀)^3^ |  | C57BL/6 WT  (*n* = 3♂ 6♀)^3^ | *Ghrh*KO  (*n* = 1♂ 5♀)^1^ |
| Body weight (g) | * | *** | *** | 21.94 | 12.61 *** |  | 26.26 (B) | 16.13 *** (B) |  | 29.20 (A) | 24.66 * (A, a) |
| Male ♂ |  | *** | ** | 24.5 ± 0.18 | 13.1 ± 0.18 *** |  | 32.0 ± 1.15 (C) | 16.7 ± 0.41 *** |  | 33.6 ± 5.01 (B) | 15.9 *** |
| Female ♀ | *** | *** | *** | 21.3 ± 0.36 | 11.8 ± 0.14 *** |  | 24.4 ± 0.75 (C) | 15.9 ± 0.54 *** (A) |  | 27.0 ± 0.23 (A) | 24.7 ± 2.26  (A, a)) |
| Thymus |  |  |  |  |  |  |  |  |  |  |  |
| Absolute weight (mg) | *** | *** | *** | 53.2 ± 4.28 | 29.6 ± 2.56 *** |  | 58.6 ± 5.61 | 29.62 ± 1.87 *** |  | 23.4 ± 2.78  (A, a)) | 29.9 ± 2.40 |
| Relative weight (mg/g of body weight) |  |  | *** | 2.5 ± 0.24 | 2.2 ± 0.25 |  | 2.3 ± 0.26 | 1.7 ± 0.12 |  | 0.9 ± 0.12 (A, a) | 1.2 ± 0.12 (B) |
| Absolute number of cells (x10^6^) |  | *** | *** | 47.3 ± 6.11 | 32.4 ± 4.56  * |  | 35.5 ± 3.14 | 20.8 ± 1.82  * (C) |  | 16.3 ± 7.90 (A, c) | 9,3 ± 1.08 (A) |
| Relative number of cells (x10^4^/mg of thymus) |  |  | *** | 84.3 ± 15.76 | 107.9 ± 18.00 |  | 62.7 ± 5.58 (B) | 76.3 ± 7.93 (A) |  | 38.7 ± 12.27 (A, a) | 45.5 ± 5.5 (A, a) |
| Spleen |  |  |  |  |  |  |  |  |  |  |  |
| Absolute weight (mg) |  | *** | *** | 79.1 ± 3.19 | 31.0 ± 2.04 *** |  | 77.7 ± 9.4 | 35.6 ± 1.86 *** |  | 97.4 ± 4.51 (B, b) | 59.2 ± 8.19 *** (A, b) |
| Relative weight (mg/g of body weight) |  | *** |  | 3.6 ± 0.15 | 2.2 ± 0.06 *** |  | 3.1 ± 0.43 | 2.2 ± 0.10 ** |  | 3.4 ± 0.25 | 2.2 ± 0.19 ** |
| Absolute number of cells (x10^6^) | ** | *** | ** | 44.8 ± 4.61 | 20.3 ± 2.05 *** |  | 51.3 ± 6.08 | 18.1 ± 1.78 *** |  | 21.5 ± 3.18 (A, a) | 18.5 ± 3.68 |
| Relative number of cells (x10^4^/mg of spleen) |  |  | *** | 56.7 ± 5.11 | 63.6 ± 3.53 |  | 52.5 ± 3.79 | 50.2 ± 4.25 (C) |  | 21.5 ± 2.22 (A, a) | 34.8 ± 7.36 (B) |
