# Supplementary material for: The Severe Deficiency of the Somatotrope GH-Releasing Hormone/Growth Hormone/Insulin-Like Growth Factor 1 Axis of Ghrh−/− Mice Is Associated With an Important Splenic Atrophy and Relative B Lymphopenia
Source: Front Endocrinol (Lausanne). 2018 Jun 6;9:296. doi: 10.3389/fendo.2018.00296 (PMC5997896; doi:10.3389/fendo.2018.00296)
Supplement: Supplementary file 3 [file table_2.DOC]

Table II. Two-way ANOVA for effects of somatotrope deficiency on frequency of thymocytes subpopulations.
Data (mean ± SEM) are representative of one^1^, two^2^ or three^3^ independent experiments.
Bonferroni significant difference between age-matched KO *vs* WT mice : *** *p* < 0.001, ** *p* < 0.01, * *p* < 0.05.
Bonferroni significant difference from 3M mice :(A) *p* < 0.001, (B) *p* < 0.01, (C) *p* < 0.05.
Bonferroni significant difference from 6M mice :(a) *p* < 0.001, (b) *p* < 0.01, (c) *p* < 0.05.
CD3+ T cells are analyzed within the CD45+ population. T cell population is divided into dpuble negative (DN, CD4-CD8-), double positive (DP, CD4+CD8+), single positive CD4 (SP, CD4+CD8-) and single positive CD8 (SP, CD4-CD8+) T cells. 20,000 CD45+ events are recorded. FoxP3+ Treg cells are studied inside the CD4+ SP population.

| Frequency | 2-way ANOVA | | | 3M | | 6M | | 18M | |
| --- | --- | --- | --- | --- | --- | --- | --- | --- | --- |
| (% of parent population) | Interaction | Strains | Age | C57BL/6 WT  (*n* = 9)*^3^* | *Ghrh*KO  (*n* = 15)*^3^* | C57BL/6 WT  (*n* = 8)*^2^* | *Ghrh*KO  (*n* = 16)*^3^* | C57BL/6 WT  (*n* = 5)*^2^* | *Ghrh*KO  (*n* = 12)*^3^* |
| DN | * | ** | *** | 3.3 ± 0.13 | 2.2 ± 0.12 *** | 3.8 ± 0.31 | 2.5 ± 0.07 *** | 3.5 ± 0.35 | 3.4 ± 0.20 (A,a) |
| DP | * |  |  | 84.8 ± 0.70 | 87.0 ± 0.36 * | 87 ± 0.83 (C) | 86.9 ± 0.29 | 86.8 ± 1.17 | 86.4 ± 0.57 (A, a) |
| SP CD4 |  |  |  | 8.8 ± 0.56 | 7.9 ± 0.25 | 7.1 ± 0.46 (C) | 7.8 ± 0.23 | 7.6 ± 1.04 | 8.0 ± 0.35 |
| SP CD8 | ** |  | *** | 3.1 ± 0.17 | 2.9 ± 0.10 | 2.1 ± 0.11 (A) | 2.8 ± 0.12 ** | 2.0 ± 0.32 (A) | 2.2 ± 0.16 (B, b) |
| Treg (as % of SP CD4) |  | *** | ** | 4.3 ± 0.39 | 5.2 ± 0.12 * | 3.8 ± 0.40 | 4.7 ± 00.42 | 4.7 ± 1.32 | 8.4 ± 0.88 ** (A, a) |
