# Supplementary material for: The Severe Deficiency of the Somatotrope GH-Releasing Hormone/Growth Hormone/Insulin-Like Growth Factor 1 Axis of Ghrh−/− Mice Is Associated With an Important Splenic Atrophy and Relative B Lymphopenia
Source: Front Endocrinol (Lausanne). 2018 Jun 6;9:296. doi: 10.3389/fendo.2018.00296 (PMC5997896; doi:10.3389/fendo.2018.00296)
Supplement: Supplementary file 4 [file table_3.DOC]

Table III. Two-way ANOVA for effects of somatotrope deficiency on frequency of lymphocytes subpopulations in spleen, LN and blood
Data (mean ± SEM) are representative of one^1^, two^2^ or three^3^ independent experiments.
Bonferroni significant difference between age-matched KO *vs* WT mice : *** *p* < 0.001, ** *p* < 0.01, * *p* < 0.05.
Bonferroni significant difference from 3M mice :(A) *p* < 0.001, (B) *p* < 0.01, (C) *p* < 0.05.
Bonferroni significant difference from 6M mice :(a) *p* < 0.001, (b) *p* < 0.01, (c) *p* < 0.05.

CD19+ B cells and CD3+ T cells are analyzed within the CD45+ population. T cell population is divided into CD4+ and CD8+ T cells. 20,000 CD45+ events are recorded. Naïve (CD44lowCD62Lhi), TCM (CD44hiCD62Lhi) and TEM (CD44hi CD62low) cells are analyzed within de CD3+CD4+ or CD3+CD8+ populations. FoxP3+ Treg cells are studied inside the CD4+ SP population.

| Frequency | 2-way ANOVA | | | 3M | | 6M | | 18M | |
| --- | --- | --- | --- | --- | --- | --- | --- | --- | --- |
| (% of parent population) | Interaction | Strains | Age | C57BL/6 WT  (*n* = 15)^1^ | *Ghrh*KO  (*n* = 16)*^1^* | C57BL/6 WT  (*n* = 8)*^2^* | *Ghrh*KO  (*n* = 16)*^1^* | C57BL/6 WT  (*n* = 9)*^3^* | *Ghrh*KO  (*n* = 6)*^1^* |
| Spleen |  |  |  |  |  |  |  |  |  |
| Bcell | * | *** | ** | 60.0 ± 1.36 | 52.5 ± 1.18 *** | 61.4 ± 0.85 | 51.7 ± 1.06 *** | 61.9 ± 1.67 | 60.8 ± 2.52 |
| Tcell | ** |  | *** | 30.6 ± 1.23 | 35.5 ± 1.17 * | 28.9 ± 1.25 | 33.9 ± 1.41* | 28.3 ± 1.84 | 22.6 ± 2.12 (A,a) |
| T CD4 | *** |  | * | 57.4 ± 0.66 | 56.2 ± 1.04 | 57.0 ± 0.40 | 54.3 ± 0.86 | 55.7 ± 1.08 | 62.9 ± 2.52 *** (A, a) |
| T CD8 | ** |  | * | 36.7 ± 0.61 | 36.9 ± 1.08 | 36.0 ± 0.67 | 38.8 ± 0.81 | 37.1 ± 0.93 | 31.4 ± 2.83 * (C, c) |
| CD4 naive | *** | *** | *** | 12.0 ± 0.62 | 29.7 ± 1.30 *** | 12.1 ± 0.75 | 21.0 ± 1.36 *** (A) | 3.4 ± 0.36  (A, a) | 9.6 ± 2.67 (A, a) |
| CD4 TCM | *** | *** | ** | 61.7 ± 2.20 | 45.6 ± 1.42 *** | 52.1 ± 2.03 (B) | 50.7 ± 1.39 | 21.2 ± 2.57 (A, a) | 21.5 ± 4.04 (A, a) |
| CD4 TEM |  | * | *** | 24.6 ± 1.62 | 23.7 ± 1.47 | 35.5 ± 2.08 (B) | 27.9 ± 1.99 | 74.7 ± 2.60 (A, a) | 68.5 ± 6.33 (A, a) |
| CD8 naive |  | *** | *** | 47.9 ± 1.05 | 57.5 ± 1.26 *** | 47.1 ± 1.74 | 59.2 ± 1.55 *** | 17.9 ± 2.18 ( A, a) | 29.8 ± 6.02 ** (A, a) |
| CD8 TCM |  | *** | *** | 40.8 ± 1.31 | 31.0 ± 1.02 *** | 42.6 ± 1.57 | 31.2 ± 1.48 *** | 59.4 ± 3.69  (A, a) | 44.5 ± 2.15 *** (A, a) |
| CD8 TEM |  |  | *** | 7.1 ± 0.69 | 7.3 ± 0.59 | 9.4 ± 0.57 | 7.6 ± 0.42* | 22.4 ± 2.89  (A, a) | 24.4 ± 4.17 |
| Treg |  |  | *** | 14.0 ± 0.38 | 17.3 ± 0.60 ** | 17.9 ± 0.69 (B) | 18.0 ± 0.61 | 29.3 ± 1.98 (A, a) | 29.2 ± 2.43  (A, a) |
| Lymph node |  |  |  |  |  |  |  |  |  |
| Bcell |  | *** | *** | 33.8 ± 1.85 | 26.5 ± 1.56 * | 39.8 ± 3.42 | 27.6 ± 1.94 ** | 53.6 ± 2.50  (A, b) | 48.1 ± 4.08  (A, a) |
| Tcell |  | ** | *** | 63.2 ± 1.82 | 70.1 ± 1.60 | 56.5 ± 3.43 | 68.2 ± 2.27 ** | 43.3 ± 2.66 (A, b) | 42.3 ± 4.04  (A, a) |
| T CD4 |  | *** | *** | 53.1 ± 0.71 | 57.8 ± 1.13 ** | 50.4 ± 0.48 | 55.5 ± 1.05 * | 42.0 ± 0.95 (A, a) | 48.4 ± 1.99 * (A, a) |
| T CD8 |  | *** | ** | 43.5 ± 0.74 | 39.0 ± 1.15** | 44.5 ± 0.38 | 40.8 ± 1.00* | 49.8 ± 1.07 ** (B, c) | 42.4 ± 2.46* |
| CD4 naive |  | *** | *** | 15.2 ± 0.97 | 35.7 ± 1.72 *** | 16.6 ± 0.66 | 31.4 ± 1.78 *** | 10.6 ± 1.33 | 24.5 ± 3.37 *** (A, c) |
| CD4 TCM | ** | *** | *** | 72.1 ± 1.80 | 48.7 ± 1.95 *** | 63.0 ± 4.68  (C) | 54.3 ± 1.70 | 52.9 ± 2.39  (A, c) | 37.6 ± 3.25 ** (B, a) |
| CD4 TEM |  |  | *** | 11.5 ± 0.99 | 13.4 ± 1.29 | 18.7 ± 4.17 | 13.3 ± 0.88 | 36.2 ± 3.01  (A, a) | 37.1 ± 5.15  (A, a) |
| CD8 naive |  | ** | *** | 57.6 ± 1.19 | 66.4 ± 1.21 ** | 59.7 ± 1.31 | 65.1 ± 1.51 | 39.5 ± 2.72  (A, a) | 40.2 ± 4.64 (A, a) |
| CD8 TCM |  | *** | *** | 37.3 ± 1.25 | 25.4 ± 0.84 *** | 34.5 ± 1.44 | 28.0 ± 1.73 | 54.1 ± 2.21 (A, a) | 47.5 ± 4.29 (A, a) |
| CD8 TEM | ** |  | *** | 2.6 ± 0.21 | 2.8 ± 0.21 | 3.5 ± 0.27 | 3.0 ± 0.25 | 5.9 ± 0.81  (A) | 9.2 ± 1.66 ** (A, a) |
| Treg |  |  | *** | 12.5 ± 0.53 | 12.5 ± 0.22 | 16.1 ± 0.51  (A) | 16.3 ± 0.70  (B) | 30.3 ± 2.13 (A, a) | 30.7 ± 2.12  (A, a) |
| Blood |  |  |  |  |  |  |  |  |  |
| Bcell | * | *** | *** | 46.1 ± 2.65 | 43.3 ± 2.55 | 54.2 ± 3.10 | 43.1 ± 5.39 | 75.7 ± 3.58  (A, b) | 51.2 ± 5.26 ** |
| Tcell |  |  | ** | 38.7 ± 1.64 | 30.4 ± 3.62* | 29.8 ± 3.83 | 33.3 ± 6.31 | 17.2 ± 2.00  (A) | 26.5 ± 3.78* |
| T CD4 |  | * | *** | 54.9 ± 1.55 | 55.3 ± 0.96 | 52.2 ± 1.94 | 49.6 ± 3.53 | 35.5 ± 2.03 (A, a) | 51.5 ± 3.38 *** |
| T CD8 | * | ** | *** | 42.5 ± 1.58 | 37.3 ± 1.43* | 44.9 ± 2.09 | 46.1 ± 2.95 (C) | 58.2 ± 2.53 (A, b) | 44.5 ± 3.79 ** |
| CD4 naive |  | *** |  | 15.8 ± 0.88 | 27.1 ± 4.20 * | 19.3 ± 1.93 | 31.9 ± 2.87 * | 18.7 ± 6.06 | 30.6 ± 4.45 |
| CD4 TCM | *** | *** | ** | 76.7 ± 1.72 | 36.6 ± 5.31 *** | 67.3 ± 2.22 | 49.9 ± 4.06 ** (C) | 51.9 ± 5.02 (A, c) | 40.9 ± 4.03 |
| CD4 TEM | ** |  | *** | 6.6 ± 0.90 | 27.8 ± 7.45 *** | 12.1 ± 1.79  (C) | 14.0 ± 2.83 | 34.9 ± 6.08  (A, b) | 28.3 ± 8.25 |
| CD8 naive | * |  |  | 53.4 ± 2.86 | 31.7 ± 6.19 ** | 48.6 ± 3.18 | 44.9 ± 4.03 | 32.5 ± 5.99 (B, c) | 38.8 ± 6.74 |
| CD8 TCM |  |  | * | 42.6 ± 3.00 | 42.8 ± 1.87 | 40.5 ± 2.03 | 41.52 ± 2.39 | 49.0 ± 5.05 | 50.5 ± 4.66 |
| CD8 TEM | ** |  |  | 2.6 ± 0.46 | 17.4 ± 5.08 * | 8.4 ± 1.81 | 8.34 ± 1.71 | 20.8 ± 6.55 (A, c) | 10.5 ± 2.94 |
| Treg |  |  | *** | 9.2 ± 0.98 | 8.0 ± 0.63 | 7.5 ± 0.90 | 7.8 ± 0.44 | 13.0 ± 0.92  (C, b) | 11.2 ± 1.99 (c) |
